# The single nucleotide variant at c.662A>G in human RRM2B is a loss‐of‐function mutation

**DOI:** 10.1002/mgg3.1497

**Published:** 2020-09-15

**Authors:** Yen‐Tzu Tseng, Shang‐Wei Li, Wei‐Chun HuangFu, Yun Yen, I‐Hsuan Liu

**Affiliations:** ^1^ Department of Animal Science and Technology National Taiwan University Taipei Taiwan; ^2^ Graduate Institute of Cancer Biology and Drug Discovery College of Medical Science and Technology Taipei Medical University Taipei Taiwan; ^3^ Ph.D. Program for Cancer Molecular Biology and Drug Discovery College of Medical Science and Technology Taipei Medical University Taipei Taiwan; ^4^ TMU Research Center of Cancer Translational Medicine Taipei Medical University Taipei Taiwan; ^5^ Cancer Center Taipei Municipal WanFang Hospital Taipei Taiwan; ^6^ Research Center for Developmental Biology and Regenerative Medicine National Taiwan University Taipei Taiwan; ^7^ School of Veterinary Medicine National Taiwan University Taipei Taiwan

## Abstract

**Background:**

Mitochondrial DNA maintenance defects (MDMDs) is one of the critical pediatric dysfunction. One of the recent report indicated that a severe patient of MDMDs carries the NP_056528.2:p.Asn221Ser (N221S) variation in the RRM2B gene (NM_015713.5). However, there is no direct evidence demonstrating the nature of the N221S variation.

**Materials and Methods:**

This study aimed to utilize zebrafish and morpholino oligomer (MO) knockdown technique to provide direct evidence for the nature of the N221S variation in the RRM2B.

**Results:**

The results showed that two distinct MOs were both able to perturb the expression of *rrm2b* in zebrafish and dose‐dependently induced morphological defects. Furthermore, co‐injection of human wild‐type *RRM2B* mRNA with MO‐e4i4 successfully rescued the developmental defects, whereas co‐injection of *RRM2B*/*N221S* mRNA with MO‐e4i4 did not rescue the developmental defects.

**Conclusion:**

In conclusion, the functional assay in this study provided the direct evidence proving that the N221S variation is a loss‐of‐function mutation and plausibly related to the pathogenic developmental defects found in the infants of previous clinical reports.

## 1 INTRODUCTION

Mitochondria are crucial for energy production in eukaryotic cells. It might be life‐threatening with mitochondrial malfunction. Mitochondrial diseases originate from pathogenic mitochondrial DNA (mtDNA) mutations that lead to defects in various mitochondrial proteins disrupting the electron transport chain and oxidative phosphorylation. With these dysfunctions, mitochondria are unable to produce sufficient energy in different tissues, especially, in the highly ATP demanding tissues such as cardiac muscle, liver, the renal, and central nervous systems (Spinazzola, 2011). Previously reported mitochondrial DNA maintenance defects (MDMDs), resulting from the pathogenic mutations in nuclear genes involved in mtDNA balancing, includes two malicious conditions: mtDNA depletion and multiple mtDNA deletion (El‐Hattab, Craigen, & Scaglia, 2017). There are two main sources of MDMDs, which are disruption of mtDNA synthesis and imbalance of the deoxyribonucleotide triphosphate (dNTPs) pool (El‐Hattab & Scaglia, 2013). Since all DNA synthesis and repair are strongly dependent on dNTPs, maintaining the dNTPs pool is critical.

Ribonucleotide reductase (RNR) is responsible for catalysis of the reduction of ribonucleotide triphosphate to dNTPs by radicals (Nordlund & Reichard, 2006). The RNR is comprised of a homodimeric large subunit (R1) as well as a homodimeric small subunit (R2). The rate‐limiting component of RNR is the R2 subunit that has a highly conserved tyrosyl residue generating the radicals by the ferric iron center (Chabes, Pfleger, Kirschner, & Thelander, 2003). Furthermore, the R2 subunit has two homologous forms. One of the forms, encoded by the gene *RRM2* (OMIM: 180390), is decomposed during mitosis. Another form, p53‐controlled RNR small subunit 2 (p53R2) (OMIM: 604712), is maintained throughout all phases of the cell cycle and is encoded by the gene *RRM2B* in human (Hakansson, Hofer, & Thelander, 2006). The stable function of RRM2B is the key to provide a copious amount of dNTP for mtDNA synthesis and repair (Kollberg et al., 2009).

There are about 31 clinical incidents reported with *RRM2B* variations in pediatric patients (Keshavan et al., 2020). Most of the patients with *RRM2B* autosomal recessive mutation at various locations died within the first few months of life exhibiting the mtDNA depletion syndrome (Keshavan et al., 2020; Kropach, Shkalim‐Zemer, Orenstein, Scheuerman, & Straussberg, 2017). Because muscle tissues and the central nervous system demand much energy, myopathy, lactic acidosis as well as encephalopathy are often observed in patients with *RRM2B* mutation (Keshavan et al., 2020; Kropach et al., 2017; Stojanovic et al., 2013). Among all the variations, a novel point missense variation: NP_056528.2:p.Asn221Ser, c.662A>G (rs863224193, Database of Single Nucleotide Polymorphisms (dbSNP), National Center for Biotechnology Information, NCBI) located in the highly conserved coding region of *RRM2B* (NM_015713) on chromosome 8 (chr8:103231064hg19) was reported recently (Penque et al., 2019). There are three isoforms of the RRM2B resulting from alternative splicing (NP_056528.2, NP_001165948.1, and NP_001165949.1). The NP_056528.2:p.Asn221Ser variation on RRM2B isoform 1 causes a variant near the two conserved iron‐binding sites that is crucial for catalysis (Penque et al., 2019). The same genomic variation point lead to RRM2B protein variation in the same conserved domain at the other two splice isoforms, NP_001165948.1:p.Asn293Ser and NP_001165949.1:p.Asn169Ser, as confirmed by the protein alignment. In this study, we used RRM2B isoform 1 (NP_056528.2, encoded by the transcript ENST00000251810) as primary target, which is the most prevalent splice variant of RRM2B in human (the GTEx Portal on 06/05/2020), and described this variant as N221S hereafter. Although a previous study indicates that other mutations located near the iron‐binding site are likely pathogenic (Bourdon et al., 2007), the effects of N221S variation are still uncertain. The clinical significance of N221S variation shown in ClinVar (NCBI; VCV000215094.2) database is still stated as conflicting interpretations of pathogenicity. There are two clinical reports from two independent families in ClinVar with accession ID: SCV000807524.1 and SCV000844949.1, respectively (Table 1). Both of the reported patients are homozygous variants in *RRM2B* with N221S. They present similar symptoms such as hearing loss and hypotonia. However, both of the two clinical reports do not have detail functional assay of the N221S variation. Currently, there is no strong evidence indicates that *RRM2B* with the N221S variation is a loss‐ofs‐function protein leading to pathogenic disease. Therefore, it is important to investigate whether N221S is pathogenic providing insights into MDMDs caused by *RRM2B* mutation as well as establishing the therapeutic foundation for the future.

**TABLE 1:** Summary of clinical evidences with *RRM2B* variation from two independent families

ClinVar Accession ID	Gender	Age	Genotype	Symptoms
SCV000807524.1	Female	4 month old	Homozygous variant	Renal tubular acidosis
				Congenital glaucoma
				Hypotonia
				Sensorineural hearing loss
SCV000844949.1	Male	3 month old	Homozygous variant	Metabolic lactic acidosis
				Hypotonia
				Sensorineural hearing loss

The crucial residues for enzyme activity of RRM2B protein are highly conserved across different species (Shang, Li, Feng, & Cui, 2011). The functions of RRM2B are also similar between human and zebrafish (Shang et al., 2011). In this study, zebrafish was used as vertebrate model to evaluate the nature of *RRM2B*/*N221S* variant. We use two morpholino oligomers (MOs) targeted at the junction between intron 2‐exon 3 (MO‐i2e3) and exon 4‐intron 4 (MO‐e4i4), respectively, to knock down the expression of *rrm2b* in zebrafish. Subsequently, in order to investigate the potential pathogenic effects of N221S variation, we used human wild‐type *RRM2B* and *RRM2B*/*N221S* mRNAs to rescue the impaired zebrafish embryos. Overall, this study aims to provide evidence for the pathogenic effects of the N221S variation on *RRM2B* gene. We proved that N221S variation on *RRM2B* gene is a loss‐ofs‐function mutation.

## 2 MATERIALS AND METHODS

### 2.1 Ethical compliance

All experimental procedures in this study were reviewed and approved by the Institutional Animal Care and Use Committee of National Taiwan University (NTU‐108‐EL‐00118) and were performed in accordance with the approved guidelines.

### 2.2 Zebrafish husbandry

The AB wild‐type zebrafish were obtained from Taiwan Zebrafish Core Facility (National Health Research Institutes) and maintained at a density of two to four fish per 3‐L tank in the aquatic facility with an automatic circulation system. The culture system was maintained at 28.5 ± 0.5°C under a light‐to‐dark cycle of 14:10 h, and the fish were fed with live adult brine shrimp twice a day (Wei & Liu, 2014). The embryos were collected after spontaneous spawning and were staged by hour postfertilization (hpf) at 28.5°C in E3 medium using morphological criteria (Kimmel, Ballard, Kimmel, Ullmann, & Schilling, 1995).

### 2.3 Gene knockdown by MOs

**FIGURE 1:**
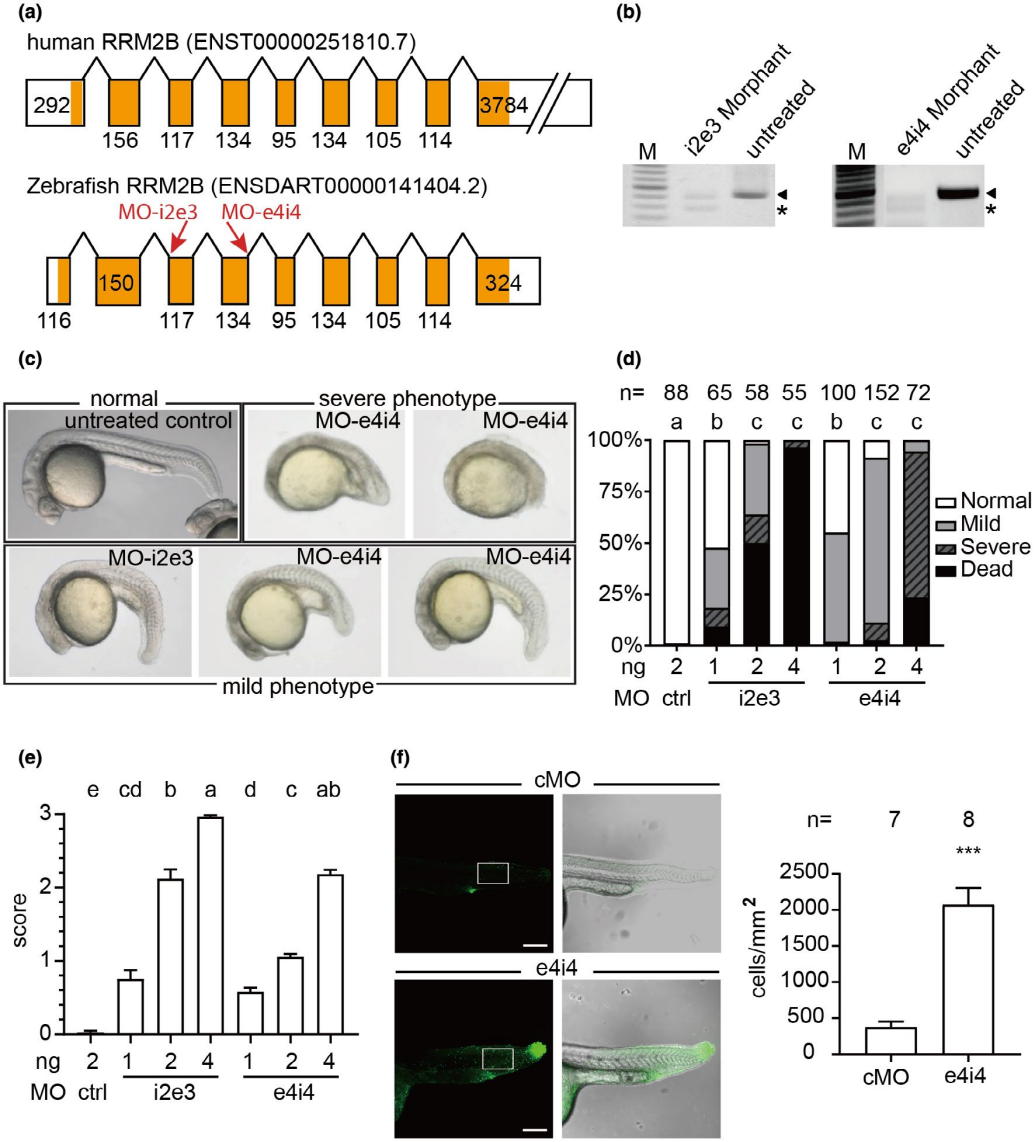
Phenotypic characterization of *rrm2b* knocked down zebrafish. (a) Comparison between human *RRM2B* and zebrafish *rrm2b* transcripts indicates a high conservation through evolution. The exons were presented as boxes and the coding regions were shaded in orange. Length of each exon was indicated at each box. The target sites for MOs are as indicated on zebrafish *rrm2b* transcripts. (b) RT‐PCR analysis of *rrm2b* from untreated control, MO‐i2e3, and MO‐e4i4 morphants indicate that both MOs targeting zebrafish *rrm2b* disrupt the maturation of *rrm2b* mRNA and result in exon skipping. (c) The micrographs of MO‐i2e3 and MO‐e4i4 morphants at 24 hpf were classified into four groups: normal, mild, severe, and dead according to the severity. (d) Penetrances of characterized phenotypes, normal, mild, severe, and dead, in MO‐control (ctrl), MO‐i2e3 (i2e3), or MO‐e4i4 (e4i4)‐treated zebrafish embryos were calculated and compared. The ratio of normal embryo was statistically analyzed. Different lowercase letters on top of the histograms represented significant differences (*p* < 0.05) among groups. (e) The morphants were scored as 0, 1, 2, and 3 for different phenotypic severity: normal, mild, severe, and death, respectively. The scores of severity were statistically analyzed. Different lowercase letters on top of the histograms represented significant differences among groups (*p* < 0.05 for statistical significance). (f) Dead cells were labeled by acridine orange emitting green fluorescence in the confocal micrographs. In MO‐control morphants, sporadic dead cells were stained with more emphasis at the posterior end of the yolk extension while MO‐e4i4 morphants showed drastically more dead cells throughout the body. The green fluorescence positive cells within the selected area were calculated (***p < 0.001)

To knockdown zebrafish rrm2b, antisense MOs were designed against intron 2‐exon 3 (MO‐i2e3) and exon 4‐intron 4 (MO‐e4i4) splicing sites (Figure 1a); additionally, a standard control MO (MO‐control) was used as the control (Gene Tools LLC, Philomath, Oregon, USA). All MOs were dissolved in distilled water to make a 2 mM stock and diluted to the desired concentration (1, 2, and 4 ng) with 0.5% phenol red (Sigma Chemical Co., St. Louis, MO, USA) before use. Microinjection was performed at the one‐cell stage in embryos with desired concentration per injection as previously described (Chang et al., 2013).

### 2.4 Morphological evaluation of zebrafish embryos

To evaluate the importance of zebrafish *rrm2b* during embryogenesis, the MOs (MO‐control, MO‐i2e3, and MO‐e4i4) were delivered to embryos (morphants) by microinjection at 0 hpf. At 24 hpf, the micrographs of morphants were documented (Leica DM2500, Wetzlar, DE). The acquired images were categorized into four classes (normal, mild, severe, and dead) according to the severity of morphological defects. Additionally, the different morphological scores for normal, mild, severe, and death were set at 0, 1, 2, and 3, respectively. The score of severity is calculated as the mean of morphological score in each treatment group. The sample size (*n*) of each group was indicated on top of histogram.

### 2.5 Acridine orange staining assay

To evaluate the cell death, 24 hpf control and MO‐e4i4 (4 ng) morphants were stained with 5 μg/ml acridine orange (Invitrogen) for 5 min followed by three washes with E3 medium. Subsequently, the embryos were subjected to confocal microscopy (TCS SP5, Leica) immediately. All images were acquired by Z projection. The total dead cells were counted with ImageJ (Rueden et al., 2017).

### 2.6 Molecular cloning and functional validation of human RRM2B

To validate the function of human RRM2B, rescue experiments were performed in *rrm2b* morphants with or without oxidative stress. The pCMV6‐entry‐h*RRM2B* and pCMV‐entry‐h*RRM2B*/*N221S* plasmids were as previously described (Penque et al., 2019). As a comparison to N221S variation, a point mutation for A61P (Keshavan et al., 2020) was introduced into h*RRM2B* to create the pCMV6‐entry‐h*RRM2B*/*A61P* plasmid. Primers used for point mutation are list in Table 2. To synthesize mRNA for microinjection, the pCMV6‐entry‐h*RRM2B*, pCMV‐entry‐h*RRM2B*/*N221S*, and pCMV‐entry‐h*RRM2B*/*A61P* were linearized by *Pci*I, and the mRNAs of *RRM2B*, *RRM2B*/*N221S* and *RRM2B*/*A61P* were subsequently synthesized using the T7 mMESSAGE mMACHINE Kit (Ambion, Austin, TX, USA). The synthesized mRNAs were aliquoted, and stored at −80°C. The mRNAs were mixed with 0.5% phenol red immediately before each experiment. For rescue experiment, 100 pg mRNA of target genes were co‐injected with 4 ng MO‐e4i4. For oxidative stress induction, embryos were cultured in E3 medium with or without the supplementation of 0.5 mM H_2_O_2_ for 8 h (Raguraman et al., 2019).

**TABLE 2:** The oligo sequences used in this study

Oligo name	Sequence 5′ → 3′
Morpholino oligos	
e4i4	TTTCATGTTCATCTCACCTCTCTTT
i2e3	TCCACCTAAAGAAAACCCATACAGT
mRNA splicing	
E2‐F	TTCCCTATTCAGTATCCAGACATC
E5‐R	CTGAAATCCACTGTAAGGCCC
Generate A61P point mutation	
A61P‐F	CCTTCCTTCTGGACAGCAGAAGAGGTC
A61P‐R	CTGTGCCTGTTTATACATTTTCCAAAT
Mitochondrial DNA quantification	
*nd1* (ENSDART00000093596.3)	GGGCACCCATACCCATGCCCTAT
	TGCGCTACAGCTCGTAAGGC
*ef1a* (NM_200009.2)	GGCCCATTTCTGGGTTCATGC
	CAACAAGTGCTTGTGCAGGGT
Mitochondrial biogenesis	
*actb1* (NM_131031.2)	TTGCTCCTTCCACCATGAAG
	CCTGCTTGCTGATCCACATC
*nd1* (ENSDART00000093596.3)	GCCTACGCCGTACCAGTATT
	GTTTCACGCCATCAGCTACTG
*nd5* (ENSDART00000093622.3)	CCTCCCATCTTAACGCCTGAGCC
	CGTAGGTCTTGTGTCGGGGGC
Oxidative stress	
*gclc* (NM_199277.2)	CTATCTGGAGAACATGGAGG
	CATTTTCCTCTGTTGACCGG
*gstp1* (NM_131734.3)	TTCAGTCCAACGCCATGC
	ATGAGATCTGATCACCAACC
*Cat* (NM130912.2)	AGATGAAACTGTGGAAGGAGGGTC
	AAACACTTTGGCTTTGGAGTAGCG

### 2.7 RNA extraction and reverse transcription PCR (RT‐PCR)

Total RNA was extracted from zebrafish embryos using TRIzol Reagent (Thermo Fisher Scientific, Waltham, MA, USA) according to the manufacturer's instruction. Ten or 15 embryos in each treatment were collected as a pool for each experiment. The single‐stranded cDNA was synthesized from 2 μg of total RNA with oligo(dT)_15_ primer and SuperScript III Reverse Transcriptase (Thermo Fisher Scientific).

In order to confirm the perturbation of mRNA splicing, primers flanking exons 2 and 5 were designed (Table 2). To perform PCR reaction, 1 μl cDNA, 2 μl specific primers (10 μM), 1 μl dNTP (10 mM), and 5 units Ex Taq DNA polymerase (Takara, Kusatsu, JP) were mixed in a total volume of 50 μl and the reactions were performed in a thermal cycler (Takara) with initial denaturation at 94°C for 5 min, 35 cycles of denaturation at 94°C for 30 s, annealing at 60°C for 10 s, extension at 72°C for 1 min, and final extension at 72°C for 7 min. PCR products were recovered from agarose gel for subsequent DNA sequencing (Center for Biotechnology, National Taiwan University, Taipei, TW).

### 2.8 Quantitative real‐time PCR (qPCR)

To quantitatively evaluate the mitochondrial DNA (mtDNA), total DNA was extracted from 8 hpf embryos using cell and tissue genomic DNA extraction kit (Geneaid, New Taipei City, TW). Briefly, embryos were homogenized and lysed by lysis buffer with proteinase K (1 mg/ml) at 60°C for 30 min. Proteins were removed by adding protein removal buffer followed by centrifugation at 14,000× *g* for 3 min. Supernatants were collected and examined by qPCR.

To quantitatively analyze the expression levels of target genes and mtDNA copy numbers, cDNA, or genomic DNA were mixed with 5 μl iQ SYBR Green Supermix (Bio‐Rad) and 1 μl of primer set mix to a total volume of 10 μl. To amplify the desired fragments of target genes, the reactions were performed at 95°C for 3 min, and then, 39 cycles of 95°C for 3 s and 60°C for 30 s followed by 60°C for 1 min with a thermal cycler (QuantStudio 3 Real‐Time PCR Systems, Thermo Fisher Scientific). The sequences of primer pairs used in this study are listed in Table 2.

### 2.9 Statistical analysis

All data were presented as mean ± standard error of the mean (SEM).The statistical analysis was performed using Prism 8 software (GraphPad, San Diego, CA, USA). The phenotypic penetrance and score were subjected to Kruskal–Wallis test with Dunn's multiple comparisons. The acridine orange stain was subjected to Mann–Whitney test. The qPCR results for mtDNA copy number and gene expressions were subjected to ordinary one‐way ANOVA with Tukey's multiple comparison test with a single pooled variance. A *p* value less than 0.05 was considered a statistically significant difference.

## 3 RESULTS AND DISCUSSION

### 3.1 Reduction of *rrm2B* expression by MO treatments‐induced developmental defects in zebrafish embryos

In order to investigate the importance of *rrm2b* during embryogenesis, we designed two MOs (MO‐i2e3 and MO‐e4i4), targeted at intron 2‐exon 3 and exon 4‐intron 4 splicing site, respectively, to knockdown the expression of zebrafish *rrm2b* (Figure 1a). MO‐i2e3 and MO‐e4i4 morphants at 24 hpf were sacrificed for RT‐PCR assay. The results showed that both MO‐i2e3 and MO‐e4i4 morphants exhibited significantly lower *rrm2b* expression compared to untreated controls with alternative PCR products (Figure 1b, alternative PCR products were indicated by asterisk). The sequencing results of the alternative PCR products indicated that both MOs perturbed the maturation of *rrm2b* mRNA and induced exon skipping. Accordingly, the formation of head and tail was significantly disrupted in the severe morphants (Figure 1c). The anterior part of the morphants was poorly developed, especially the part of the heart that required high mitochondrial activity. The ratio of normal embryos in each group was significantly reduced in a dose‐dependent manner (Figure 1d). Additionally, each morphant was scored as 0, 1, 2, and 3 according to its severity of the phenotype (normal, mild, severe, and dead). Statistical analysis on the scores of severity also indicated that the phenotypic severity induced by both MOs were increased dose‐dependently (Figure 1e). Subsequently, the acridine orange staining assay revealed that significantly higher number of dead cells can be found in MO‐e4i4 morphants comparing to MO‐control (Figure 1f). These results showed that MOs designed in this study successfully knocked down the expression of *rrm2b* causing development defects and abnormal cell death.

Because there is an energy shift from glycolysis to oxidative phosphorylation in the human after birth, the phenotypes of defective *RRM2B* usually appear soon after birth (Nsiah‐Sefaa & McKenzie, 2016). Many clinical reports show that mutation of *RRM2B* causes myopathy, lactic acidosis, and ultimately death usually in infancy or early childhood (Finsterer & Zarrouk‐Mahjoub, 2018; Keshavan et al., 2020; Kropach et al., 2017; Penque et al., 2019). In these patients, the mutation of *RRM2B* leads to MDMDs with insufficient mtDNA; therefore, inadequate mitochondrial activity crippled cells demanding high energy such as muscle and renal cells (Chen et al., 2019; El‐Hattab et al., 2017). In contrast to mammals, the major fetal energy in oviparous animals depends on the yolk. The avian and fish eggs are generally believed to contain very little carbohydrate and rich in free amino acids and lipids. Therefore, it is reasonable to observe phenotypes in early zebrafish *rrm2b* morphants. Consistent to this speculation, the *rrm2b*‐targeting MO‐treated zebrafish exhibited developmental defects and died in the early stages of development (Figure 1c). Additionally, one study shows that silencing the expression of *RRM2B* induced the mitochondrial membrane depolarization and induction of ROS leading to premature senescence in young fibroblasts (Kuo et al., 2012). Similarly, *rrm2b* knockdown by MOs in the present study showed that many cells die prematurely providing evidence to the development defects caused by abnormal *RRM2B* function (Figure 1f). Taken together, the results in this study indicated that knocking down the *rrm2b* in zebrafish embryos is fatal in neonatal stages and adversely affected the morphogenesis. This implied the crucial role of *RRM2B* in development can be related to the death of infants who had mutation of *RRM2B* in clinical reports (Finsterer & Zarrouk‐Mahjoub, 2018).

### 3.2 Human *RRM2B* mRNA with N221S or A61P mutation cannot rescue embryonic defects and mitochondrial dysfunction

**FIGURE 2:**
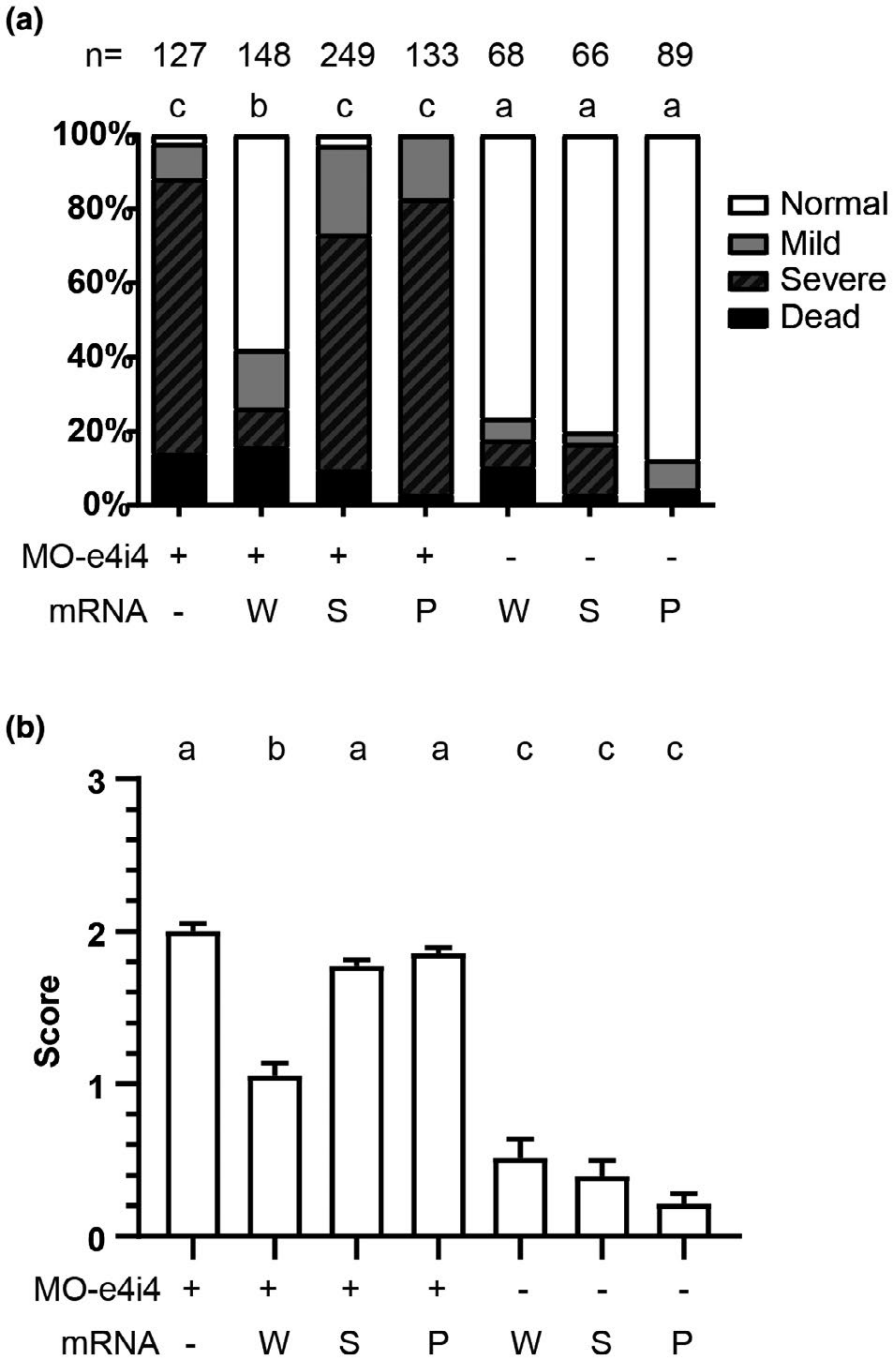
Rescuing effects of human wild‐type *RRM2B*, *RRM2B*/*N221S*, or *RRM2B*/*A61P* in MO‐e4i4‐treated zebrafish embryos. (a) The zebrafish embryos were microinjected with MO‐e4i4 with or without the supplementations of mRNAs encoding for human wild‐type *RRM2B* (W), *RRM2B*/*N221S* (S), or *RRM2B*/*A61P* (P). The ratio of normal embryo was statistically analyzed. (b) The morphants were scored as 0, 1, 2, and 3 for different phenotypic severity: normal, mild, severe, and death, respectively. The scores of severity were statistically analyzed. Different lowercase letters on top of the histograms represented significant differences among groups (*p* < 0.05 for statistical significance)

In principle, if human *RRM2B* functions identically as zebrafish *rrm2b*, ectopically overexpress human *RRM2B* should, at least partially, rescue the developmental defects in *rrm2b* morphants. As expected, human *RRM2B* mRNA significantly rescued the penetrance of severe phenotype from 74% to 10.2% (Figure 2a). In contrast, *RRM2B*/*N221S* mRNA did not exert similar rescuing effects compared to *RRM2B* (Figure 2a). As a negative control, a recently reported pathogenic mutation *RRM2B*/*A61P* (NP_056528.2:p.Ala61Pro) mRNA (Keshavan et al., 2020) also showed a comparable rescuing effect as *RRM2B*/*N221S* (Figure 2a). Similarly, the phenotypic scores also indicated that phenotypic severity induced by MO‐e4i4 was rescued by *RRM2B* but not *RRM2B*/*N221S* or *RRM2B*/*A61P* (Figure 2b).

**FIGURE 3:**
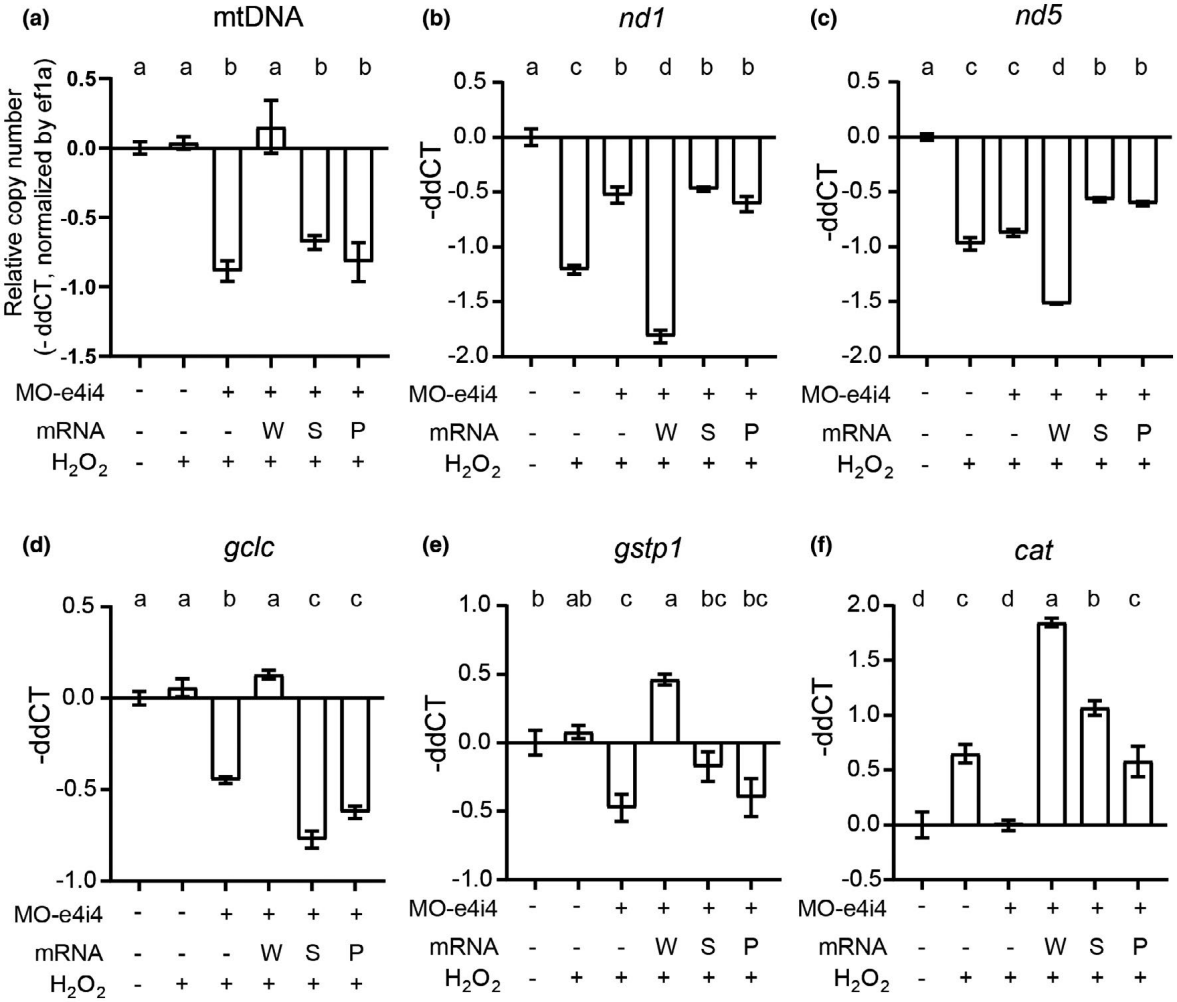
*Rrm2b* plays a conservative role in mitochondrial homeostasis in response to oxidative stress. (a) Mitochondrial DNA (mtDNA) abundance were examined under oxidative stress induction (0.5 mM H_2_O_2_). The zebrafish embryos were microinjected with MO‐e4i4 with or without the supplementations of mRNAs encoding for human wild‐type *RRM2B* (W), *RRM2B*/*N221S*, (S) or *RRM2B*/*A61P* (P). Total DNA was extracted from 8 hpf morphants and mtDNA was semiquantitatively determined by qPCR with mtDNA specific primer (*nd1*) and nuclear DNA specific primer (*ef1a*). (b‐f) Transcriptional expression levels of *nd1* (b), *nd5* (c), *gclc* (d), *gstp1* (e), and *cat* (f) were quantified by qPCR. Relative gene expression levels fold change (−ΔΔCT) were normalized by *actb1* and untreated control embryos. Different lowercase letters on top of the histograms represented significant differences (*p* < 0.05) among groups

*RRM2B* gene is crucial in mtDNA synthetization, and highly conserved between human and zebrafish (Figure 1a) (Bourdon et al., 2007). A previous study showed that *rrm2b* in zebrafish is also associated with DNA repair and synthesis as in mammalian cells (Shang et al., 2011). To verify whether the mtDNA synthetization was also affected, mtDNA were quantitatively evaluated in MO‐e4i4 morphants with or without the human *RRM2B* variants. Under oxidative stress, mtDNA decreased in MO‐e4i4 morphants compared with untreated embryo at 8 hpf. Human *RRM2B* mRNA effectively rescued the reduced mtDNA in MO‐e4i4 morphants, but not *RRM2B*/*N221S* or *RRM2B*/*A61P* mRNA (Figure 3a). These results indicated that the N221S variation perturbed the normal function of RRM2B.

Previous studies indicated that RRM2B modulate mitochondrial homeostasis in response to oxidative stress by turning down electron transport chain (Cho et al., 2015) and scavenging reactive oxygen species (ROS) (Kang et al., 2013; Liu, Xue, & Yen, 2008). To further investigate the role of *rrm2b* in zebrafish early embryogenesis, expression levels of mitochondrial biogenesis (*nd1* and *nd5*) and ROS‐scavenging enzymes and responders (*gclc*, *gstp1* and *cat*) were examined by qPCR. Under oxidative stress, expression levels of *nd1* and *nd5* were significantly reduced in 8 hpf zebrafish embryos, but the reduction in *nd1* level was significantly ameliorated in MO‐e4i4 morphants (Figure 3b,c) suggesting zebrafish *rrm2b* play a role in modulating mitochondrial biogenesis in response to oxidative stress. Furthermore, human *RRM2B* mRNA not only significantly rescued the reduction of *nd1* expression but also further lower the express of *nd5* (Figure 3b,c). In contrast, both *RRM2B*/*N221S* and *RRM2B*/*A61P* variants failed to exert any modulatory effect on *nd1* and *nd5* (Figure 3b,c).

Under oxidative stress, a series of transcriptional responses will be triggered. Although the oxidative responders (*gclc* and *gstp1*) in early zebrafish embryos (Hahn et al., 2014) were not significantly triggered under H_2_O_2_ stimulation, the ROS scavenger enzyme catalase (*cat*) was significantly increased (Figure 3d‐f). Interestingly, all the expression levels of these panel markers were significantly reduced in MO‐e4i4 morphants compared to the H_2_O_2_‐treated controls (Figure 3d‐f) indicating *rrm2b* play a critical role in responding to oxidative stress. Similarly, human *RRM2B* mRNA not only significantly rescued the expression levels of *gclc* and *gstp1* but also further boosted the express of *catalase*, while both *RRM2B*/*N221S* and *RRM2B*/*A61P* variants failed to exert similar rescuing effect on the oxidative stress response (Figure 3d‐f). Taken together, these results indicate that zebrafish *rrm2b* play a role in mitochondrial homeostasis and oxidative response in early zebrafish embryos just like its counterparts in mammalian cells. Human *RRM2B* are able to rescue MO‐e4i4 morphants phenotype penetrance in correlation with the mitochondrial homeostasis and oxidative response.

Recently, accumulating evidence shows that *RRM2B* variants are highly related to MDMDs. It is suggested that patients expressing serious lactic acidosis, muscle hypotonia, ataxia, and renal tubulopathy may have *RRM2B* mutations (Finsterer & Zarrouk‐Mahjoub, 2018). The X‐ray crystal structure of the RRM2B reveal that there are two conserved iron‐binding sites and one active site (Smith et al., 2009). The N221S mutation is located in one of the conserved iron‐binding sites in the RRM2B. There is one clinical report indicating a patient with a novel *RRM2B* variant, N221S, has a series of severe MDMDs including hypotonia and lactic acidosis (Penque et al., 2019). Although this clinical report suggests the potential relation between N221S and MDMDs, the N221S mutation still has not been confirmed as pathogenic due to lack of direct evidence from the patient (Nordlund & Reichard, 2006). At a similar position, a patient with NP_056528.2:p.Ile224Ser, which has been confirmed as a pathogenic, homozygous mutation exhibit serious myopathy and mtDNA depletion in muscle (Bornstein et al., 2008). Both of the patients cannot properly develop and failed to thrive at the early stage of infancy. Similarly, in our results, MO‐e4i4 morphants with *RRM2B*/*N221S* mRNA still exhibited severe developmental defects and mitochondrial dysfunction (Figures 2 and 3). These results indicate the importance of the conserved iron‐binding sites in the *RRM2B* gene and the mutations within these regions are highly pathogenic.

In conclusion, the present study demonstrated that *rrm2b* plays a crucial role in zebrafish embryogenesis. With this model, this study provided direct evidence confirming that the N221S variation disrupts the function of *RRM2B* gene product and the N221S variation is a loss‐of‐function mutation.

## CONFLICT OF INTEREST

The authors have no conflict of interest to declare.

## AUTHOR CONTRIBUTION

Yen‐Tzu Tseng performed most of the experiments and participate in the manuscript drafting and revision. Shang‐Wei Li participated in some experiments and data analysis, and drafted the original manuscript. Wei‐Chun HuangFu contributed to the experimental design and manuscript drafting. Yun Yen contributed to the research conception and experimental design. I‐Hsuan Liu contributed to the research conception, experimental design, as well as manuscript drafting and revision. All the authors proofread and approved the submitted manuscript.
